# Supplementary material for: Optimizing Sample Size for Population Genomic Study in a Global Invasive Lady Beetle, Harmonia Axyridis
Source: Insects. 2020 May 9;11(5):290. doi: 10.3390/insects11050290 (PMC7291016; doi:10.3390/insects11050290)
Supplement: Supplementary file 1 [file insects-11-00290-s001.zip › Table S1.docx]

| Sample | Raw reads | Enzyme reads | Alignment reads | Target number (3<depth<500) |
| --- | --- | --- | --- | --- |
| LNSY1 | 6457053 | 5477918 | 1842756 | 41821 |
| LNSY2 | 6457053 | 5708335 | 2090923 | 41670 |
| LNSY3 | 6457053 | 5745786 | 1925708 | 40691 |
| LNSY4 | 6457053 | 5644519 | 2049337 | 41295 |
| LNSY5 | 6457053 | 5515663 | 2026565 | 41711 |
| LNSY6 | 6851047 | 6107914 | 2065406 | 40635 |
| LNSY7 | 6851047 | 6157565 | 2157577 | 41234 |
| LNSY8 | 6851047 | 6150814 | 1957875 | 40924 |
| LNSY9 | 6851047 | 6029621 | 2183322 | 41519 |
| LNSY10 | 6851047 | 5875047 | 2118117 | 41443 |
| LNSY11 | 9585714 | 8697750 | 3147274 | 41303 |
| LNSY12 | 9585714 | 8903456 | 3318187 | 41801 |
| LNSY13 | 9585714 | 8718033 | 2795273 | 41076 |
| LNSY14 | 9585714 | 8640801 | 2913976 | 42181 |
| LNSY15 | 9585714 | 8673757 | 3004801 | 42197 |
| LNSY16 | 10288931 | 9431967 | 3409691 | 41441 |
| LNSY17 | 10288931 | 9395763 | 3409116 | 41423 |
| LNSY18 | 10288931 | 9490316 | 3168463 | 41303 |
| LNSY19 | 10288931 | 9423214 | 3336540 | 42245 |
| LNSY20 | 10288931 | 9357693 | 3472256 | 42198 |
| PLKK1 | 6869065 | 6030867 | 2254737 | 40523 |
| PLKK2 | 6869065 | 6019436 | 2261797 | 41380 |
| PLKK3 | 6869065 | 5916110 | 1661137 | 40241 |
| PLKK4 | 6869065 | 5979225 | 1439654 | 40649 |
| PLKK5 | 6869065 | 5803127 | 1979830 | 41722 |
| PLKK6 | 7193220 | 6498806 | 1635890 | 40437 |
| PLKK7 | 7193220 | 6214826 | 2080219 | 41817 |
| PLKK8 | 7193220 | 5716922 | 2010419 | 41736 |
| PLKK9 | 7193220 | 6400128 | 2341120 | 41166 |
| PLKK10 | 7193220 | 6281640 | 2255279 | 41789 |
| PLKK11 | 9544374 | 8673108 | 3212208 | 41131 |
| PLKK12 | 9544374 | 8342846 | 3076048 | 42370 |
| PLKK13 | 9544374 | 8247567 | 3010277 | 41958 |
| PLKK14 | 9544374 | 8407630 | 2944569 | 42562 |
| PLKK15 | 9544374 | 8345198 | 3243912 | 42726 |
| PLKK16 | 12072403 | 10614343 | 3220779 | 42996 |
| PLKK17 | 12072403 | 10604178 | 3056548 | 43712 |
| PLKK18 | 12072403 | 10846484 | 2718633 | 42878 |
| PLKK19 | 12072403 | 10879658 | 3619498 | 43664 |
| PLKK20 | 12072403 | 10602883 | 4147482 | 44037 |

Table S1 Sequencing details of *Harmonia axyridis* in LNSY and PLKK populations.
