# Supplementary material for: Optimizing Sample Size for Population Genomic Study in a Global Invasive Lady Beetle, Harmonia Axyridis
Source: Insects. 2020 May 9;11(5):290. doi: 10.3390/insects11050290 (PMC7291016; doi:10.3390/insects11050290)
Supplement: Supplementary file 1 [file insects-11-00290-s001.zip › Table S3.docx]

Table 2 Mean and 95% confidence interval (CI) of estimated parameters in resampling replicates in LNSY and PLKK populations

|  | LNSY | | | | | |  | PLKK | | | | | |  | LNSY/PLKK | |
| --- | --- | --- | --- | --- | --- | --- | --- | --- | --- | --- | --- | --- | --- | --- | --- | --- |
| Replicates | *Ae* | | *Ho* | | *uHe* | |  | *Ae* | | *Ho* | | *uHe* | |  | *Fst* | |
|  | Mean | 95% CI | Mean | 95% CI | Mean | 95% CI |  | Mean | 95% CI | Mean | 95% CI | Mean | 95% CI |  | Mean | 95% CI |
| x10 | 1.1017 | 1.0986  1.1048 | 0.0740 | 0.0718  0.0762 | 0.0777x | 0.0752  0.0802 |  | 1.1133 | 1.1102  1.1164 | 0.0805 | 0.0777  0.0833 | 0.0854 | 0.0833  0.0875 |  | 0.0492 | 0.0364  0.0620 |
| x20 | 1.1025 | 1.0995  1.1055 | 0.0746 | 0.0725  0.0767 | 0.0783 | 0.0761  0.0805 |  | 1.1117 | 1.1094  1.1140 | 0.0796 | 0.0782  0.0810 | 0.0845 | 0.0828  0.0862 |  | 0.0556 | 0.0420  0.0692 |
| x30 | 1.1047 | 1.1015  1.1079 | 0.0767 | 0.0738  0.0796 | 0.0802 | 0.0775  0.0829 |  | 1.1112 | 1.1091  1.1133 | 0.0803 | 0.0786  0.0820 | 0.0845 | 0.0832  0.0858 |  | 0.0488 | 0.0393  0.0583 |
| x40 | 1.1030 | 1.0987  1.1073 | 0.0751 | 0.0716  0.0786 | 0.0790 | 0.0758  0.0822 |  | 1.1123 | 1.1100  1.1146 | 0.0807 | 0.0781  0.0833 | 0.0849 | 0.0830  0.0868c |  | 0.0366 | 0.0249  0.0483 |
| x50 | 1.1043 | 1.1020  1.1066 | 0.0763 | 0.0743  0.0783 | 0.0799 | 0.0783  0.0815 |  | 1.1126 | 1.1110  1.1142 | 0.0808 | 0.0790  0.0826 | 0.0849 | 0.0838  0.0860 |  | 0.0206 | 0.0117  0.0295 |
| x60 | 1.1039 | 1.0995  1.1083 | 0.0759 | 0.0714  0.0804 | 0.0796 | 0.0760  0.0832 |  | 1.1096 | 1.1074  1.1118 | 0.0791 | 0.0772  0.0810 | 0.0828 | 0.0813  0.0843 |  | 0.0398 | 0.0265  0.0531 |
| x70 | 1.1070 | 1.1036  1.1104 | 0.0775 | 0.0740  0.0810 | 0.0817 | 0.0790  0.0844 |  | 1.1111 | 1.1090  1.1132 | 0.0804 | 0.0790  0.0818 | 0.0840 | 0.0827  0.0853 |  | 0.0583 | 0.0488  0.0678 |
| x80 | 1.1035 | 1.1014  1.1056 | 0.0750 | 0.0733  0.0767 | 0.0789 | 0.0776  0.0802 |  | 1.1111 | 1.1079  1.1143 | 0.0796 | 0.0769  0.0823 | 0.0837 | 0.0813  0.0861 |  | 0.0325 | 0.0209  0.0441 |
| x90 | 1.1035 | 1.1008  1.1062 | 0.0764 | 0.0735  0.0793 | 0.0793 | 0.0769  0.0817 |  | 1.1114 | 1.1083  1.1145 | 0.0800 | 0.0780  0.0820 | 0.0842 | 0.0820  0.0864 |  | 0.0367 | 0.0312  0.0422 |
| x100 | 1.1006 | 1.0970  1.1042 | 0.0731 | 0.0703  0.0759 | 0.0771 | 0.0745  0.0797 |  | 1.1121 | 1.1089  1.1153 | 0.0803 | 0.0781  0.0825 | 0.0848 | 0.0825  0.0871 |  | 0.0347 | 0.0286  0.0408 |
