# Supplementary material for: Optimizing Sample Size for Population Genomic Study in a Global Invasive Lady Beetle, Harmonia Axyridis
Source: Insects. 2020 May 9;11(5):290. doi: 10.3390/insects11050290 (PMC7291016; doi:10.3390/insects11050290)
Supplement: Supplementary file 1 [file insects-11-00290-s001.zip › Table S4.docx]

Table 3 Mean and 95% confidence interval (CI) of estimated parameters in resampling individuals in LNSY and PLKK populations

|  | LNSY | | | | | |  | PLKK | | | | | |  | LNSY/PLKK | |
| --- | --- | --- | --- | --- | --- | --- | --- | --- | --- | --- | --- | --- | --- | --- | --- | --- |
| Individuals | *Ae* | | *Ho* | | *uHe* | |  | *Ae* | | *Ho* | | *uHe* | |  | *Fst* | |
|  | Mean | 95% CI | Mean | 95% CI | Mean | 95% CI |  | Mean | 95% CI | Mean | 95% CI | Mean | 95% CI |  | Mean | 95% CI |
| n2 | 1.0934 | 1.0911  1.0958 | 0.0739 | 0.0721  0.0757 | 0.0772 | 0.0755  0.0789 |  | 1.0997 | 1.0976  1.1017 | 0.0793 | 0.0776  0.0810 | 0.0816 | 0.0801  0.0831 |  | 0.0524 | 0.0458  0.0589 |
| n4 | 1.1071 | 1.1056  1.1085 | 0.0764 | 0.0750  0.0777 | 0.0805 | 0.0793  0.0817 |  | 1.1149 | 1.1139  1.1159 | 0.0803 | 0.0793  0.0813 | 0.0848 | 0.0842  0.0855 |  | 0.0394 | 0.0353  0.0434 |
| n6 | 1.1079 | 1.1066  1.1091 | 0.0752 | 0.0743  0.0761 | 0.0800 | 0.0791  0.0808 |  | 1.1165 | 1.1155  1.1174 | 0.0795 | 0.0788  0.0802 | 0.0847 | 0.0842  0.0853 |  | 0.0370 | 0.0345  0.0396 |
| n8 | 1.1086 | 1.1077  1.1095 | 0.0755 | 0.0747  0.0763 | 0.0802 | 0.0795  0.0809 |  | 1.1176 | 1.1168  1.1183 | 0.0801 | 0.0795  0.0807 | 0.0850 | 0.0844  0.0856 |  | 0.0373 | 0.0360  0.0385 |
| n10 | 1.1089 | 1.1080  1.1098 | 0.0754 | 0.0747  0.0760 | 0.0803 | 0.0797  0.0809 |  | 1.1180 | 1.1176  1.1184c | 0.0800 | 0.0796  0.0804 | 0.0850 | 0.0847  0.0852 |  | 0.0381 | 0.0370  0.0391 |
| n15 | 1.1097 | 1.1093  1.1102 | 0.0758 | 0.0754  0.0762 | 0.0807 | 0.0804  0.0811 |  | 1.1185 | 1.1182  1.1189 | 0.0795 | 0.0792  0.0799 | 0.0849 | 0.0847  0.0852 |  | 0.0394 | 0.0388  0.0400 |
